# Synthesis and crystal structure of poly[(3-amino-1,2,4-triazole)(μ_3_-1*H*-benzimidazole-5,6-di­carboxyl­ato)cobalt(II)]

**DOI:** 10.1107/S2056989021005867

**Published:** 2021-06-15

**Authors:** Chen Zhao, Yi Li, Jin-Sheng Xiao, Peng-Dan Zhang, Xue-Qian Wu, Qiang Chen

**Affiliations:** a Beijing Key Laboratory for Green Catalysis and Separation and Department of Environmental Chemical Engineering, Faculty of Environment and Life, Beijing University of Technology, Beijing 100124, People’s Republic of China

**Keywords:** crystal structure, coordination polymer, mixed-ligands synthetic strategy, imidazole, di­carb­oxy­lic acid

## Abstract

The asymmetric unit of the title coordination polymer, [Co(C_9_H_4_N_2_O_4_)(C_2_H_4_N_4_)]_*n*_ or [Co(*L*_1_)(*L*_2_)]_*n*_, consists of one crystallographically independent Co^2+^ centre, one *L*_1_^2−^ ligand and one *L*_2_ ligand (*L*_1_ = 1*H*-benzimidazole-5,6-di­carb­oxy­lic acid, *L*_2_ = 3-amino-1,2,4-triazole). The Co^2+^ centre is coordinated by two carboxyl­ato-*O* atoms from two independent *L*_1_^2−^ ligands and two nitro­gen atoms from *L*_2_ and another *L*_1_ ligand. Thus, the metal center adopts a four-coordinate mode, forming a tetra­hedral geometry. Inter­estingly, through the combination of two *L*_1_^2−^, two *L*_2_ ligands and two Co^2+^ ions, a basic repeating unit is constructed, resulting in the formation of a one-dimensional straight chain structure. These chains are further expanded to the final three-dimensional framework *via* N—H⋯O hydrogen-bonding inter­actions.

## Abstract 2

The structure of the title coordination polymer is determined by multiple hydrogen-bonding inter­actions. In the crystal, two ligands coordinate with the metal centre, generating chains, which are further connected with each other *via* N—H⋯O hydrogen bonds, and expanded into the final framework through additional N—H⋯O hydrogen bonds.

## Chemical context   

Over the past two decades, coordination polymers (CPs) have been demonstrated to represent a new type of crystalline organic–inorganic hybrid materials, and are unique in terms of their potentially high porosities, tunable pores, and diverse compositions (Du *et al.*, 2013 ▸; Kitagawa *et al.*, 2007 ▸; Cui *et al.*, 2016 ▸). These features have enabled CPs to be constructed with great potential for various applications, such as gas adsorption/separation (Zhao *et al.*, 2018 ▸), chemical sensing (Huang *et al.*, 2017 ▸), heterogeneous catalysis (He *et al.*, 2020 ▸) and energy storage/conversion (Lu *et al.*, 2020 ▸). Meanwhile, the crystalline nature of CPs allows for the accurate determination of their structures using X-ray diffraction techniques and further, the revealing of structure–property relationships. The great potential of these compounds certainly promotes the development of synthetic strategies for new CPs. It has been demonstrated that many efficient synthetic routes, including metal exchange (Wang *et al.*, 2017 ▸), ligand substitution (Han *et al.*, 2014 ▸), directional construction based on secondary building units (SBUs) (Zou *et al.*, 2016 ▸), and topology-guided reticular chemistry principles (Wang *et al.*, 2016 ▸) have shown some advantages in fabricating new CPs with multiple structures and functionalities. In addition to the methods mentioned above, the mixed-ligands strategy is also considered to be an important approach for the integration of the properties of related ligands into a single coordination polymer and hence expansion of the structural diversity of CPs (Macreadie *et al.*, 2020 ▸). In this context, we report the synthesis and crystal structure of the title coordination polymer poly[(3-amino-1,2,4-triazole)(μ_3_-1*H*-benzimidazole-5,6-di­carboxyl­ato)cobalt(II)] (**1**), which was prepared by the solvothermal method using two simple ligands and a cobalt salt.
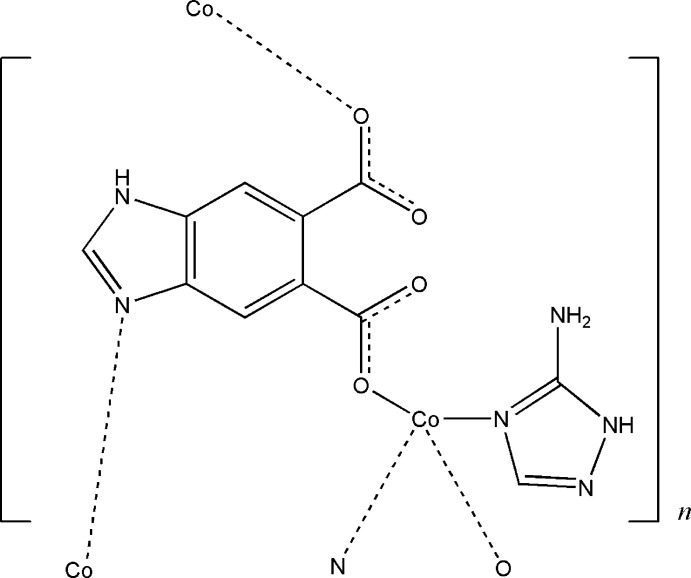


## Structural commentary   

**Figure 1:**
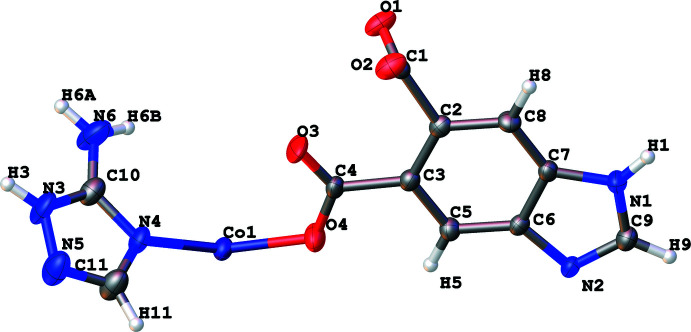
A view of the asymmetric unit of the title coordination polymer showing the atom numbering with displacement ellipsoids drawn at the 50% probability level.

**Figure 2:**
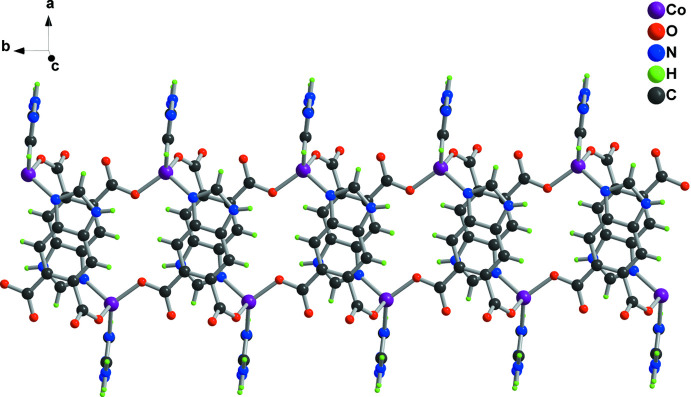
A view of the one-dimensional straight chain structure within the coordination polymer.

**Figure 3:**
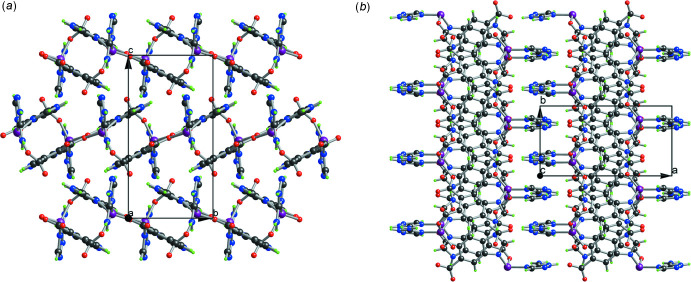
Structure of the title coordination polymer viewed along the (*a*) *a* axis and (*b*) *c* axis, respectively.

The title coordination polymer (**1**) crystallizes in the monoclinic system, *P*2_1_/*c* space group, and its asymmetric unit contains one Co^2+^ center, one *L*_1_^2−^ anion and one *L*_2_ ligand (Fig. 1 ▸). The metal center adopts a typical tetra­hedral linkage geometry to coordinate with two carboxyl­ato-*O* atoms from two independent *L*_1_^2−^ ligands and two nitro­gen atoms, one from *L*_2_ and another from an *L*_1_ ligand. Inter­estingly, through the combination of two *L*_1_^2−^, two *L*_2_ ligands and two Co^2+^ ions, a basic repeating unit is constructed, resulting in the formation of a one-dimensional straight chain structure (as shown in Fig. 2 ▸). These chains are further connected *via* hydrogen bonding inter­actions (Fig. 3 ▸), generating a three-dimensional framework.

## Supra­molecular features   

**Figure 4:**
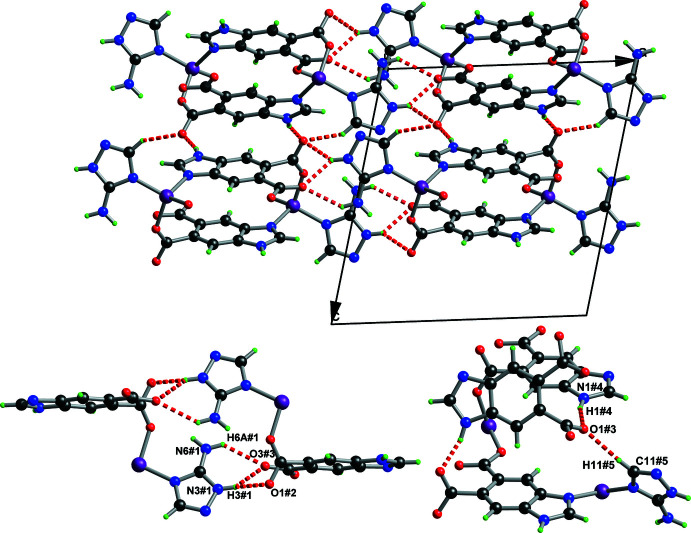
A view of the hydrogen bonds in the title coordination polymer. Intra­molecular hydrogen bonds are omitted for clarity [symmetry codes: (#1) −1 + *x*, *y*, *z*; (#2) 1 − *x*, 1 − *y*, 1 − *z*; (#3) −*x*, 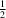 + *y*, 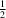 − *z*; (#4) −1 + *x*, 1 + *y*, *z*; (#5) −*x*, 1 − *y*, 1 − *z*.

As mentioned above, extensive hydrogen-bonding inter­actions in the crystal of the title coordination polymer are observed, the numerical values of which are presented in Table 1 ▸. As shown in Fig. 4 ▸, each chain is linked to adjacent chains by N1—H1⋯O1 hydrogen bonds into infinite layer structures parallel to the *bc* plane. Meanwhile, these layers are linked by other inter­molecular hydrogen bonds (*e.g*., N3—H3⋯O3 and N6—H6*A*⋯O3), resulting in the formation of the final three-dimensional supra­molecular network. Due to the regular distribution of Co^2+^ metal sites, the high density of nitro­gen atoms in the structure, and the packing arrangement of the supra­molecular network, the coordination polymer has the potential to work as a mol­ecular catalyst or to serve as the precursor material for preparing an electrocatalyst.

## Database survey   

A search of the Cambridge Crystallographic Database (CSD version 5.42, update of Feb 2021, Groom *et al.*, 2016 ▸) for structures with 1*H*-benzimidazole-5,6-di­carboxyl­ate gave 372 hits of which some are coordination polymers with prominent free pore space (also known as metal-organic frameworks, MOFs). For example, Li and co-workers reported a new three-dimensional non-inter­penetrating metal–organic framework (BARKUD01), featuring one-dimensional nanotube channels and exhibiting excellent gas separation performances (Li *et al.*, 2017 ▸). There are some Co^2+^ complexes containing only ligand *L*_1_ [refcodes AJIKIO (Fu *et al.*, 2009 ▸), NUCGUO (Wei *et al.*, 2009 ▸), ROMRUH (Xu *et al.*, 2009 ▸), ROMRUH01 (Wei *et al.*, 2009 ▸), ROMRUH02 (Shi *et al.*, 2012 ▸), SILZAP (Lo *et al.*, 2007 ▸), SOGCEX (Gao *et al.*, 2008 ▸), and YOTFET (Song *et al.*, 2009 ▸)]. However, none of these exhibit a tetra­hedral geometry around the Co atom. A zinc complex (BOVQUZ; Li *et al.*, 2009 ▸) displays a tetra­hedral coordination around the metal center. By using cyclo­penta­dienyliron dicarbonyl dimer as a starting material, two new Fe^II^-based MOFs have been constructed (HOHBEN and HOHBIR; Li *et al.*, 2014 ▸). As a typical imidazole-carboxyl­ate ligand, 1*H*-benzimidazole-5,6-di­carboxyl­ate could bind rare earth/transition-metal centers with multiple coordination modes, which provides an ideal platform for the preparation of various coordination polymers, such as BASTOG (Sun *et al.*, 2010 ▸), EHETAO (Jin *et al.*, 2016 ▸) and FELBAC (Chai *et al.*, 2018 ▸).

## Synthesis and crystallization   

A mixture of Co(NO_3_)_2_·6H_2_O (20 mg, 0.069 mmol), 1*H*-benzimidazole-5,6-di­carb­oxy­lic acid (10 mg, 0.049 mmol), 3-amino-1,2,4-triazole (10 mg, 0.119 mmol), DMA (2 mL) and H_2_O (2 mL) were added to a 20 mL vial. The reaction system was then heated at 373 K for 72 h in an oven. Purple block-shaped crystals of the title compound suitable for X-ray analysis were obtained.

## Refinement   

Crystal data, data collection and structure refinement details are summarized in Table 2 ▸. H atoms were placed in calculated positions (N—H = 0.86 Å, C—H = 0.93 Å) and refined as riding with *U*_iso_(H) = 1.2*U*_eq_(N,C)

## Supplementary Material

Crystal structure: contains datablock(s) I. DOI: 10.1107/S2056989021005867/dj2027sup1.cif

Supporting information file. DOI: 10.1107/S2056989021005867/dj2027Isup3.cdx

Structure factors: contains datablock(s) I. DOI: 10.1107/S2056989021005867/dj2027Isup4.hkl

CCDC reference: 1996100

Additional supporting information:  crystallographic information; 3D view; checkCIF report

## Figures and Tables

**Table 1:** Hydrogen-bond geometry (Å, °)

*D*—H⋯*A*	*D*—H	H⋯*A*	*D*⋯*A*	*D*—H⋯*A*
N1—H1⋯O1^i^	0.86	1.84	2.675 (4)	163
N3—H3⋯O1^ii^	0.86	2.48	3.104 (4)	130
N3—H3⋯O3^ii^	0.86	2.08	2.811 (4)	142
N6—H6*A*⋯O3^ii^	0.86	2.35	3.036 (5)	136
N6—H6*B*⋯O2^iii^	0.86	2.23	2.946 (5)	141
C5—H5⋯O4^iv^	0.93	2.58	3.312 (4)	136
C9—H9⋯N5^v^	0.93	2.48	3.332 (5)	152
C11—H11⋯O1^vi^	0.93	2.41	3.215 (5)	145

Symmetry codes: (i) -x+1, y-{\script{1\over 2}}, -z+{\script{1\over 2}}; (ii) -x+2, -y+1, -z+1; (iii) x, y+1, z; (iv) -x+1, -y+1, -z+1; (v) x-1, -y+{\script{1\over 2}}, z-{\script{1\over 2}}; (vi) x, -y+{\script{1\over 2}}, z+{\script{1\over 2}}.

**Table 2:** Experimental details

Crystal data	
Chemical formula	[Co(C_9_H_4_N_2_O_4_)(C_2_H_4_N_4_)]
*M*_r_	347.16
Crystal system, space group	Monoclinic, *P*2_1_/*c*
Temperature (K)	279
*a*, *b*, *c* (Å)	13.3368 (8), 6.8727 (4), 13.6015 (10)
β (°)	103.478 (7)
*V* (Å^3^)	1212.38 (14)
*Z*	4
Radiation type	Mo *K*α
μ (mm^−1^)	1.45
Crystal size (mm)	0.06 × 0.05 × 0.04

Data collection	
Diffractometer	Rigaku Oxford Diffraction SuperNova, Dual, Cu at home/near, AtlasS2
Absorption correction	Multi-scan (*CrysAlis PRO*; Rigaku OD, 2018 ▸)
*T*_min_, *T*_max_	0.979, 1.000
No. of measured, independent and observed [*I* > 2σ(*I*)] reflections	5618, 2475, 1839
*R*_int_	0.046
(sin θ/λ)_max_ (Å^−1^)	0.625

Refinement	
*R*[*F*^2^ > 2σ(*F* ^2^)], *wR*(*F* ^2^), *S*	0.047, 0.098, 1.03
No. of reflections	2475
No. of parameters	199
H-atom treatment	H-atom parameters constrained
Δρ_max_, Δρ_min_ (e Å^−3^)	0.45, −0.43

Computer programs: *CrysAlis PRO* (Rigaku OD, 2018 ▸), *SHELXT* (Sheldrick, 2015*a* ▸), *SHELXL* (Sheldrick, 2015*b* ▸) and *OLEX2* (Dolomanov *et al.*, 2009 ▸).

## supplementary crystallographic information

### Crystal data

**Table:**

[Co(C_9_H_4_N_2_O_4_)(C_2_H_4_N_4_)]	*F*(000) = 700
*M**_r_* = 347.16	*D*_x_ = 1.902 Mg m^−^^3^
Monoclinic, *P*2_1_/*c*	Mo *K*α radiation, λ = 0.71073 Å
*a* = 13.3368 (8) Å	Cell parameters from 1697 reflections
*b* = 6.8727 (4) Å	θ = 4.3–26.7°
*c* = 13.6015 (10) Å	µ = 1.45 mm^−^^1^
β = 103.478 (7)°	*T* = 279 K
*V* = 1212.38 (14) Å^3^	Block, purple
*Z* = 4	0.06 × 0.05 × 0.04 mm

### Data collection

**Table:**

Rigaku Oxford Diffraction SuperNova, Dual, Cu at home/near, AtlasS2 diffractometer	2475 independent reflections
Graphite monochromator	1839 reflections with *I* > 2σ(*I*)
Detector resolution: 10.3376 pixels mm^-1^	*R*_int_ = 0.046
phi and ω scans	θ_max_ = 26.4°, θ_min_ = 3.3°
Absorption correction: multi-scan (CrysAlis PRO; Rigaku OD, 2018)	*h* = −14→16
*T*_min_ = 0.979, *T*_max_ = 1.000	*k* = −8→8
5618 measured reflections	*l* = −17→12

### Refinement

**Table:**

Refinement on *F*^2^	Primary atom site location: dual
Least-squares matrix: full	Hydrogen site location: inferred from neighbouring sites
*R*[*F*^2^ > 2σ(*F*^2^)] = 0.047	H-atom parameters constrained
*wR*(*F*^2^) = 0.098	*w* = 1/[σ^2^(*F*_o_^2^) + (0.0319*P*)^2^ + 0.6634*P*] where *P* = (*F*_o_^2^ + 2*F*_c_^2^)/3
*S* = 1.03	(Δ/σ)_max_ = 0.001
2475 reflections	Δρ_max_ = 0.45 e Å^−^^3^
199 parameters	Δρ_min_ = −0.43 e Å^−^^3^
0 restraints	

### Special details

**Table:**

Geometry. All esds (except the esd in the dihedral angle between two l.s. planes) are estimated using the full covariance matrix. The cell esds are taken into account individually in the estimation of esds in distances, angles and torsion angles; correlations between esds in cell parameters are only used when they are defined by crystal symmetry. An approximate (isotropic) treatment of cell esds is used for estimating esds involving l.s. planes.

### Fractional atomic coordinates and isotropic or equivalent isotropic displacement parameters (Å^2^)

**Table:**

	*x*	*y*	*z*	*U*_iso_*/*U*_eq_	
Co1	0.75360 (3)	0.69451 (6)	0.52745 (4)	0.02172 (16)	
O2	0.76917 (18)	−0.1847 (3)	0.40067 (19)	0.0298 (6)	
O1	0.74157 (18)	0.0171 (3)	0.27211 (19)	0.0303 (6)	
O4	0.66113 (18)	0.4785 (3)	0.4920 (2)	0.0328 (6)	
O3	0.77486 (18)	0.2754 (4)	0.4537 (2)	0.0418 (7)	
N2	0.3154 (2)	0.1240 (4)	0.3922 (2)	0.0203 (6)	
N1	0.3399 (2)	−0.1552 (4)	0.3188 (2)	0.0254 (7)	
H1	0.326047	−0.266046	0.289464	0.030*	
N4	0.9004 (2)	0.6928 (4)	0.6053 (2)	0.0264 (7)	
C4	0.6862 (3)	0.3188 (5)	0.4544 (3)	0.0216 (8)	
C7	0.4351 (2)	−0.0655 (5)	0.3432 (2)	0.0197 (7)	
C6	0.4188 (2)	0.1110 (5)	0.3892 (2)	0.0190 (7)	
C8	0.5319 (2)	−0.1215 (5)	0.3322 (3)	0.0223 (8)	
H8	0.541882	−0.239926	0.302707	0.027*	
N3	1.0667 (2)	0.7206 (5)	0.6511 (3)	0.0402 (9)	
H3	1.129713	0.736543	0.647408	0.048*	
C1	0.7158 (3)	−0.0546 (5)	0.3456 (3)	0.0210 (8)	
C3	0.5986 (2)	0.1848 (5)	0.4132 (2)	0.0205 (7)	
C9	0.2731 (3)	−0.0376 (5)	0.3493 (3)	0.0254 (8)	
H9	0.203572	−0.067057	0.340978	0.031*	
C2	0.6129 (2)	0.0051 (5)	0.3665 (2)	0.0198 (7)	
C5	0.5008 (2)	0.2361 (5)	0.4240 (3)	0.0215 (8)	
H5	0.490570	0.353422	0.454314	0.026*	
N5	1.0361 (2)	0.6888 (5)	0.7393 (3)	0.0449 (9)	
N6	0.9921 (3)	0.7490 (5)	0.4760 (3)	0.0491 (10)	
H6A	1.051093	0.765185	0.461830	0.059*	
H6B	0.936808	0.749236	0.428430	0.059*	
C10	0.9863 (3)	0.7234 (5)	0.5721 (3)	0.0300 (9)	
C11	0.9367 (3)	0.6734 (6)	0.7073 (3)	0.0380 (10)	
H11	0.893449	0.650912	0.750793	0.046*	

### Atomic displacement parameters (Å^2^)

**Table:**

	*U*^11^	*U*^22^	*U*^33^	*U*^12^	*U*^13^	*U*^23^
Co1	0.0156 (3)	0.0225 (3)	0.0273 (3)	−0.0014 (2)	0.00539 (19)	−0.0020 (2)
O2	0.0248 (13)	0.0346 (14)	0.0315 (15)	0.0118 (12)	0.0097 (11)	0.0126 (12)
O1	0.0273 (14)	0.0316 (14)	0.0369 (16)	0.0087 (12)	0.0178 (12)	0.0121 (12)
O4	0.0234 (14)	0.0270 (14)	0.0481 (17)	−0.0055 (12)	0.0083 (12)	−0.0114 (13)
O3	0.0146 (13)	0.0519 (18)	0.061 (2)	−0.0066 (13)	0.0135 (13)	−0.0209 (15)
N2	0.0151 (14)	0.0249 (15)	0.0214 (16)	−0.0029 (13)	0.0057 (12)	−0.0010 (13)
N1	0.0206 (15)	0.0259 (16)	0.0307 (18)	−0.0053 (13)	0.0080 (13)	−0.0088 (13)
N4	0.0142 (14)	0.0321 (17)	0.0327 (18)	−0.0010 (13)	0.0047 (13)	0.0032 (14)
C4	0.0197 (18)	0.0233 (18)	0.0228 (19)	−0.0069 (15)	0.0068 (14)	−0.0014 (15)
C7	0.0200 (18)	0.0199 (17)	0.0197 (18)	−0.0035 (15)	0.0055 (14)	−0.0002 (14)
C6	0.0175 (17)	0.0200 (17)	0.0201 (18)	−0.0007 (15)	0.0060 (14)	0.0020 (14)
C8	0.0229 (18)	0.0186 (17)	0.026 (2)	0.0030 (16)	0.0069 (15)	−0.0033 (15)
N3	0.0159 (16)	0.062 (2)	0.043 (2)	−0.0036 (16)	0.0083 (15)	0.0089 (18)
C1	0.0222 (18)	0.0175 (17)	0.0239 (19)	−0.0018 (15)	0.0067 (15)	−0.0053 (15)
C3	0.0184 (17)	0.0233 (18)	0.0199 (18)	−0.0012 (15)	0.0046 (14)	0.0005 (15)
C9	0.0190 (18)	0.0292 (19)	0.028 (2)	−0.0019 (16)	0.0052 (15)	−0.0012 (16)
C2	0.0178 (17)	0.0223 (18)	0.0187 (18)	0.0024 (15)	0.0030 (14)	0.0041 (15)
C5	0.0203 (18)	0.0196 (17)	0.0240 (19)	−0.0011 (15)	0.0041 (15)	−0.0003 (15)
N5	0.0260 (18)	0.068 (3)	0.039 (2)	−0.0059 (17)	0.0032 (16)	0.0083 (19)
N6	0.0297 (19)	0.084 (3)	0.038 (2)	0.0013 (19)	0.0161 (16)	0.010 (2)
C10	0.0228 (19)	0.031 (2)	0.038 (2)	0.0027 (17)	0.0099 (17)	0.0063 (18)
C11	0.026 (2)	0.055 (3)	0.034 (2)	−0.001 (2)	0.0066 (17)	0.010 (2)

### Geometric parameters (Å, º)

**Table:**

Co1—O2^i^	1.968 (2)	C7—C8	1.390 (4)
Co1—O4	1.919 (2)	C6—C5	1.384 (4)
Co1—N2^ii^	2.016 (3)	C8—H8	0.9300
Co1—N4	1.997 (3)	C8—C2	1.381 (5)
O2—C1	1.272 (4)	N3—H3	0.8600
O1—C1	1.234 (4)	N3—N5	1.372 (5)
O4—C4	1.287 (4)	N3—C10	1.331 (5)
O3—C4	1.222 (4)	C1—C2	1.521 (5)
N2—C6	1.392 (4)	C3—C2	1.422 (5)
N2—C9	1.319 (4)	C3—C5	1.392 (5)
N1—H1	0.8600	C9—H9	0.9300
N1—C7	1.380 (4)	C5—H5	0.9300
N1—C9	1.337 (4)	N5—C11	1.300 (5)
N4—C10	1.342 (5)	N6—H6A	0.8600
N4—C11	1.366 (5)	N6—H6B	0.8600
C4—C3	1.490 (5)	N6—C10	1.339 (5)
C7—C6	1.405 (5)	C11—H11	0.9300
			
O2^i^—Co1—N2^ii^	111.64 (11)	N5—N3—H3	124.4
O2^i^—Co1—N4	100.15 (11)	C10—N3—H3	124.4
O4—Co1—O2^i^	107.41 (11)	C10—N3—N5	111.1 (3)
O4—Co1—N2^ii^	105.49 (11)	O2—C1—C2	119.0 (3)
O4—Co1—N4	128.46 (11)	O1—C1—O2	122.3 (3)
N4—Co1—N2^ii^	103.38 (11)	O1—C1—C2	118.6 (3)
C1—O2—Co1^iii^	130.8 (2)	C2—C3—C4	122.0 (3)
C4—O4—Co1	123.3 (2)	C5—C3—C4	118.4 (3)
C6—N2—Co1^ii^	129.5 (2)	C5—C3—C2	119.6 (3)
C9—N2—Co1^ii^	124.0 (2)	N2—C9—N1	113.5 (3)
C9—N2—C6	104.9 (3)	N2—C9—H9	123.2
C7—N1—H1	126.4	N1—C9—H9	123.2
C9—N1—H1	126.4	C8—C2—C1	115.8 (3)
C9—N1—C7	107.3 (3)	C8—C2—C3	121.5 (3)
C10—N4—Co1	129.0 (3)	C3—C2—C1	122.6 (3)
C10—N4—C11	103.3 (3)	C6—C5—C3	119.4 (3)
C11—N4—Co1	127.6 (3)	C6—C5—H5	120.3
O4—C4—C3	115.0 (3)	C3—C5—H5	120.3
O3—C4—O4	123.5 (3)	C11—N5—N3	102.1 (3)
O3—C4—C3	121.4 (3)	H6A—N6—H6B	120.0
N1—C7—C6	105.3 (3)	C10—N6—H6A	120.0
N1—C7—C8	132.6 (3)	C10—N6—H6B	120.0
C8—C7—C6	122.1 (3)	N3—C10—N4	108.5 (3)
N2—C6—C7	109.0 (3)	N3—C10—N6	124.8 (4)
C5—C6—N2	131.2 (3)	N6—C10—N4	126.7 (3)
C5—C6—C7	119.8 (3)	N4—C11—H11	122.5
C7—C8—H8	121.2	N5—C11—N4	115.1 (4)
C2—C8—C7	117.5 (3)	N5—C11—H11	122.5
C2—C8—H8	121.2		
			
Co1—O4—C4—O3	13.1 (5)	C7—C8—C2—C1	−175.4 (3)
Co1—O4—C4—C3	−168.4 (2)	C5—C3—C2—C8	−0.6 (5)
C9—N1—C7—C8	178.0 (4)	C4—C3—C2—C8	177.4 (3)
C9—N1—C7—C6	−0.2 (4)	C5—C3—C2—C1	175.7 (3)
C9—N2—C6—C5	−179.1 (4)	C4—C3—C2—C1	−6.3 (5)
Co1^ii^—N2—C6—C5	−13.5 (5)	O1—C1—C2—C8	98.3 (4)
C9—N2—C6—C7	−0.3 (4)	O2—C1—C2—C8	−77.6 (4)
Co1^ii^—N2—C6—C7	165.4 (2)	O1—C1—C2—C3	−78.3 (4)
N1—C7—C6—C5	179.3 (3)	O2—C1—C2—C3	105.8 (4)
C8—C7—C6—C5	0.8 (5)	N2—C6—C5—C3	178.5 (3)
N1—C7—C6—N2	0.3 (4)	C7—C6—C5—C3	−0.2 (5)
C8—C7—C6—N2	−178.2 (3)	C2—C3—C5—C6	0.1 (5)
N1—C7—C8—C2	−179.2 (3)	C4—C3—C5—C6	−177.9 (3)
C6—C7—C8—C2	−1.2 (5)	C10—N3—N5—C11	0.2 (4)
Co1^iii^—O2—C1—O1	173.7 (2)	N5—N3—C10—N6	−179.1 (4)
Co1^iii^—O2—C1—C2	−10.5 (4)	N5—N3—C10—N4	−0.3 (4)
O3—C4—C3—C5	174.7 (3)	C11—N4—C10—N3	0.3 (4)
O4—C4—C3—C5	−3.8 (5)	Co1—N4—C10—N3	176.1 (2)
O3—C4—C3—C2	−3.3 (5)	C11—N4—C10—N6	179.1 (4)
O4—C4—C3—C2	178.2 (3)	Co1—N4—C10—N6	−5.1 (6)
C6—N2—C9—N1	0.2 (4)	N3—N5—C11—N4	0.0 (5)
Co1^ii^—N2—C9—N1	−166.5 (2)	C10—N4—C11—N5	−0.2 (5)
C7—N1—C9—N2	0.0 (4)	Co1—N4—C11—N5	−176.1 (3)
C7—C8—C2—C3	1.1 (5)		

Symmetry codes: (i) *x*, *y*+1, *z*; (ii) −*x*+1, −*y*+1, −*z*+1; (iii) *x*, *y*−1, *z*.

### Hydrogen-bond geometry (Å, º)

**Table:**

*D*—H···*A*	*D*—H	H···*A*	*D*···*A*	*D*—H···*A*
N1—H1···O1^iv^	0.86	1.84	2.675 (4)	163
N3—H3···O1^v^	0.86	2.48	3.104 (4)	130
N3—H3···O3^v^	0.86	2.08	2.811 (4)	142
N6—H6*A*···O3^v^	0.86	2.35	3.036 (5)	136
N6—H6*B*···O2^i^	0.86	2.23	2.946 (5)	141
C5—H5···O4^ii^	0.93	2.58	3.312 (4)	136
C5—H5···O4	0.93	2.37	2.698 (4)	100
C9—H9···N5^vi^	0.93	2.48	3.332 (5)	152
C11—H11···O1^vii^	0.93	2.41	3.215 (5)	145

Symmetry codes: (i) *x*, *y*+1, *z*; (ii) −*x*+1, −*y*+1, −*z*+1; (iv) −*x*+1, *y*−1/2, −*z*+1/2; (v) −*x*+2, −*y*+1, −*z*+1; (vi) *x*−1, −*y*+1/2, *z*−1/2; (vii) *x*, −*y*+1/2, *z*+1/2.
